# Causes of Dorsal Cutaneous Branch of the Ulnar Nerve Neuropathy Among Patients Undergoing Electrodiagnostic Studies: A Series of 14 Patients

**DOI:** 10.7759/cureus.38162

**Published:** 2023-04-26

**Authors:** Lisa B Shields, Vasudeva G Iyer, Yi Ping Zhang, Christopher B Shields

**Affiliations:** 1 Norton Neuroscience Institute, Norton Healthcare, Louisville, USA; 2 Clinical Neurophysiology, Neurodiagnostic Center of Louisville, Louisville, USA

**Keywords:** electromyography, nerve conduction studies, electrodiagnostic studies, dorsal cutaneous branch of the ulnar nerve, neurosurgery, neurology

## Abstract

Background: Isolated neuropathy of the dorsal cutaneous branch of the ulnar nerve (DCBUN) is rare and most cases are secondary to trauma, often iatrogenic. The topography of sensory abnormalities and abnormal electrodiagnostic (EDX) findings are crucial in confirming DCBUN neuropathy.

Materials and methods: This is a retrospective study of patients with isolated involvement of the DCBUN from among patients referred for EDX studies for upper extremity symptoms. All patients underwent a focused neurological examination followed by EDX studies. Ultrasound (US) studies were performed in two patients.

Results: Of the 14 patients with DCBUN neuropathy, decreased pinprick sensation in the distribution of the DCBUN was noted in 11 (78%) patients. DCBUN sensory nerve action potential (SNAP) was not recordable in 13 (92%) patients. In one patient who had a recordable SNAP, the latency was prolonged, and the amplitude was decreased. Four (28%) patients had incidental EDX abnormalities suggestive of entrapment of the median nerve at the carpal tunnel. The most common cause of DCBUN neuropathy was trauma in 13 (92%) patients, of which eight were iatrogenic. No specific etiology was detected in one patient (7%). Of the two patients who underwent US studies, one had increased cross-sectional area (CSA) at the wrist with prominent fascicles and hyperechoic scar tissue, while the CSA was normal in the other patient.

Conclusions: Although rare, DCBUN neuropathy can be readily confirmed by typical clinical features and EDX findings. Surgeons should be aware of the anatomy and clinical features of DCBUN neuropathy and avoid injuring the nerve during surgical procedures at the wrist and forearm.

## Introduction

Neuropathy of the dorsal cutaneous branch of the ulnar nerve (DCBUN) may result in numbness, dysesthesia, and pain of the dorsoulnar aspect of the hand, dorsum of the fifth finger, and dorsoulnar aspect of the fourth finger [1,2]. In 1922 Stopford reported the first two cases of neuropathy of the DCBUN from a “wristlet watch” [3]. The pathogenesis and clinical presentation are similar to Wartenberg’s syndrome characterized by a focal neuropathy of the superficial radial nerve that leads to pain and paresthesia of the dorsoradial aspect of the hand [3-5].

Arising from the medial cord of the brachial plexus (fibers of the ventral rami of the C8 and T1 spinal nerve roots), the ulnar nerve runs distally through the axilla medial to the axillary artery [2,6,7]. The DCBUN leaves the main nerve trunk of the ulnar nerve and pierces the antebrachial fascia 5-10 cm proximal to the tip of the ulnar styloid process [1,4,8-15]. The DCBUN passes between the flexor carpi ulnaris tendon and ulna, courses volar to the ulnar head, and gives off two to three branches at the fifth metacarpal joint [1,12,15,16]. The DCBUN has a variable origin in the forearm and may arise from either the proximal, middle, or most commonly the distal third of the forearm [8]. The higher origin of the DCBUN makes it more susceptible to superficial injuries and lacerations [7,8]. Communications between the DCBUN and the superficial terminal branch of the ulnar nerve and superficial radial nerve can lead to variable patterns of sensory loss in injuries to the DCBUN [7,17].

Several studies in the literature have investigated the anatomy of the DCBUN, with a particular emphasis on the variations of its origin [2,8-10,14,15,18-20]. The variability of the origin and branching patterns of the DCBUN underscore the absence of a reliable surgical safe zone on the dorsum of the hand, which increases its vulnerability to iatrogenic injury during open or arthroscopic surgeries [6-8,15,18-22]. These studies concur that detailed knowledge of the DCBUN’s anatomical course is valuable in preventing iatrogenic injuries during surgeries involving the ulnar side of the wrist, when evaluating chronic pain of the dorsal aspect of the hand, and during focal nerve blocks [2,6,7,9,10]. 

Due to its superficial location, the DCBUN is susceptible to laceration, blunt trauma, or iatrogenic injuries, the latter following open reduction and internal fixation of ulnar fractures, ulnar osteotomy, ulnar reconstruction, ulnar lengthening and shortening procedures, a forearm flap, carpal tunnel release, treatment of chronic osteomyelitis, and wrist arthroscopic surgeries [1,2,7,8,20,23]. If injured, the DCBUN may give rise to a painful neuroma [2,18,21]. Vigilant dissection within 2.5 cm of the ulnar styloid process is recommended due to the close proximity of the DCBUN [14]. Additionally, it is suggested to avoid setting the 6U portal under traction during wrist arthroscopic surgery with the arm passively pronated [23]. There are a few case reports that have described the clinical features of a DCBUN injury [4,10,13,16,24,25]. 

In this study we describe the clinical features of 14 patients with DCBUN neuropathy along with the electrodiagnostic (EDX) study findings. Ultrasound (US) study of nerves has been available more recently in our facility, and only two of these patients underwent US studies. We discuss the clinical and EDX criteria to diagnose this condition and the precautions necessary to avoid iatrogenic injury to this nerve. 

## Materials and methods

We performed a 10-year (February 9, 2012- August 19, 2022) retrospective analysis of patients referred to our Neurodiagnostic Center for EDX studies to evaluate the presence of upper limb neuropathy to identify patients with isolated DCBUN neuropathy. All the patients underwent neurological examination of the upper extremities followed by nerve conduction velocity (NCV) and EMG studies. The EDX studies were performed in our American Association of Neuromuscular & Electrodiagnostic Medicine (AANEM)-accredited facility using standard protocol of our laboratory [26]. The EDX study of the DCBUN was done according to the technique described by Jabre [11]. An US study was also conducted using the GE Logiq E system and 8-18 MHz probe tracing the DCBUN. The cross-sectional area (CSA) of the nerve was measured at different sites along the course of the nerve. 

**Figure 1:**
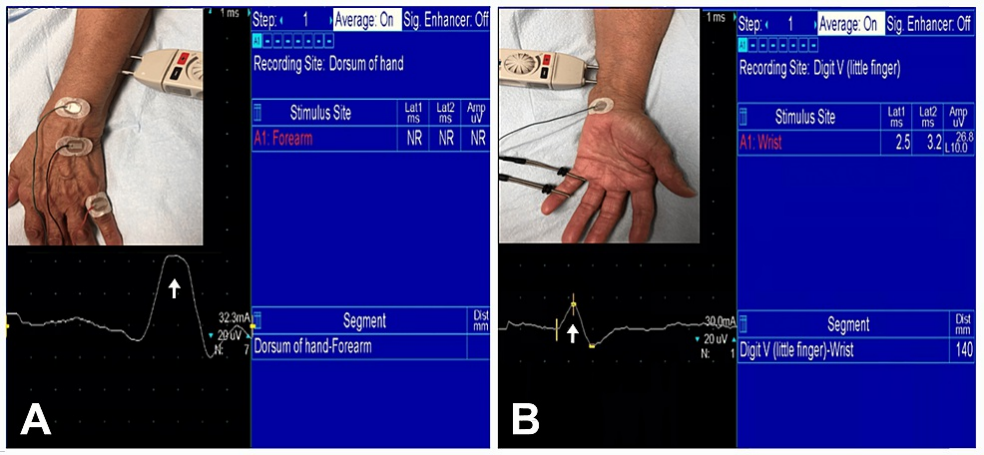
Left Ulnar Nerve Sensory Conduction Study (A) No sensory nerve action potentials (SNAP) over the dorsal cutaneous branch (recorded with surface electrodes on the dorsum) by stimulation of the ulnar nerve at the medial distal forearm. The negative waveform (arrow) is volume conducted compound muscle action potentials (CMAP) from the hypothenar muscles. (B) Normal SNAP (arrow) over the digital branch (recorded with ring electrodes around the small finger) by stimulation of the ulnar nerve at the medial wrist.

The inclusion criteria for the clinical diagnosis of DCBUN neuropathy were the following: burning pain, paresthesia, or numbness and objective sensory loss (hypoesthesia/anesthesia) or allodynia in the distribution of the DCBUN. The inclusion criteria for confirmation of DCBUN neuropathy by EDX studies included: absent sensory nerve action potentials (SNAP) or delayed peak latency, and/or reduced SNAP amplitude of DCBUN with normal SNAP in the digital branch of the little finger (Figure 1A, 1B).

A detailed history was obtained from each patient with a history of trauma. Only those patients who were sure that the sensory symptoms (numbness/paresthesia/dysesthesia in the distribution of DCBUN) occurred immediately after the surgical procedure were included in the iatrogenic group. The criterion to include patients with selective involvement of the DCBUN (based on absent/abnormal conduction in the DCBUN and normal conduction in the more distal superficial branch of the ulnar nerve as explained in Figure 1A, 1B) renders specificity to the localization to the DCBUN. All the patients had nerve conduction studies of other nerves of the upper extremities to rule out polyneuropathy/mononeuritis multiplex. Several metrics were collected including age, gender, symptom laterality (right/left), clinical history, findings on neurological examination, EDX findings, and US features. 

Informed consent was obtained from all patients. The University of Louisville IRB determined that our study was exempt according to 45 CFR 46.101(b) under Category 4. The IRB number is 22.1046. 

## Results

Clinical findings and neurological examination

A total of 14 patients were diagnosed with DCBUN neuropathy based on the presenting symptoms, findings on focused neurological examination, and EDX studies (Table 1).

**Table 1:** Demographics, Presenting Symptoms, Neurological Examination, and EDX Findings of Patients with Neuropathy of the Dorsal Cutaneous Branch of the Ulnar Nerve Who Were Evaluated at Our Neurodiagnostic Center DCBUN: dorsal cutaneous branch of the ulnar nerve; R: right; L: left; EDX: electrodiagnostic; APB: abductor pollicis brevis; NR: not recordable; SNAP: sensory nerve action potential; MVA: motor vehicle accident; MP: metacarpophalangeal; TFCC: triangular fibrocartilage complex; CTS: carpal tunnel syndrome Normal EDX values DCBUN SNAP: Latency ≤ 2.6 ms Amplitude ≥ 8 uV NR: Not recordable Asterisk: Abnormal value

Patient #	Age (years)/ Gender	Side (R/L)	Cause	Presenting Symptoms at EDX	Neurological Examination at EDX	DCBUN SNAP Latency/Amplitude	Additional EDX diagnosis
1	72/F	R	Injury: knife wound	Paresthesia dorsal hand	Decreased pinprick sensation dorsal hand	NR	Bilateral CTS
2	27/F	R	Injury to distal ulna during MVA	Hypersensitivity dorsal hand, pain/paresthesia hand	Allodynia dorsal hand, decreased pinprick sensation all digits	NR	Ipsilateral CTS
3	54/M	R	Iatrogenic: Ulnar shortening surgery	Pain forearm/ ulnar 2 digits/dorsum hand	Hyperalgesia dorsum of hand ulnar 2 digits, ulnar/dorsum palm	NR	Ipsilateral CTS
4	18/F	R	Injury: Laceration medial wrist (glass cut)	Pain/paresthesia hand	Decreased pinprick sensation medial dorsum hand/thumb	NR	None
5	44/M	R	Iatrogenic: Ulnar shortening surgery	Pain/swelling hand/dorsal wrist	Decreased pinprick sensation over dorsal medial hand	NR	None
6	62/M	R	Injury: Dog bite	Pain elbow to middle finger	Swelling dorsal hand/knuckles/IP joints; limitation of movement MP joint index finger/middle finger; decreased pinprick sensation all 4 digits/dorsum hand	NR	Ipsilateral CTS
7	48/M	R	Iatrogenic: Excision of ganglion cyst at dorsum of wrist	Numbness dorsum hand	Decreased pinprick sensation dorsum hand	NR	None
8	55/F	L	Injury to distal ulna during MVA	Burning sensation ulnar forearm; pain with wrist movements	Tenderness ulnar styloid process	NR	None
9	51/F	L	Iatrogenic: Scheker prosthesis after fracture ulna	Paresthesia dorsum hand	Normal	NR	None
10	40/F	R	Iatrogenic: Repair of torn ligament ulnar wrist	Swelling/pain/ paresthesia hand; decreased grip strength	Tenderness over ulnar wrist; decreased pinprick sensation over dorsomedial hand	NR	None
11	33/F	R	Idiopathic	Pain/paresthesia dorsum hand/ulnar 2 digits	Decreased pinprick sensation dorsal hand	3.2*, 6.3*	None
12	44/F	R	Iatrogenic: Multiple surgeries for TFCC tear	Paresthesia ulnar 2 digits/dorsum hand	Decreased pinprick sensation dorsal medial hand	NR	None
13	39/F	L	Iatrogenic: Repair of torn ligaments medial wrist	Paresthesia ulnar 2 digits, pain dorsum hand	Decreased pinprick sensation medial dorsal hand	NR	None
14	48/M	L	Iatrogenic: Wrist and forearm surgery for injury to the wrist	Pain upper extremity (“neuropathic” and “bone” pain)	Decreased pinprick sensation dorsum hand/thumb	NR	None

The mean age was 45 years (range: 18-72 years), and the majority (nine; 64%) of patients were female. The DCBUN neuropathy occurred on the right side in 10 (71%) patients and on the left in four (29%). 

All patients had pain, numbness, and/or paresthesia of the wrist and/or hand. On neurological examination, decreased pinprick sensation of the hand was noted in 11 (78%) patients. No muscle weakness of the upper extremities was detected. 

Electrodiagnostic studies

The findings of the EDX studies are summarized in Table 1. SNAPs of the DCBUN were not recordable in 13 (92%) patients. One patient had a recordable SNAP, the latency was delayed, and the amplitude was decreased. Four (28%) patients had evidence of median nerve neuropathy at the carpal tunnel, unilateral in three and bilateral in one. 

Ultrasound studies

**Figure 2:**
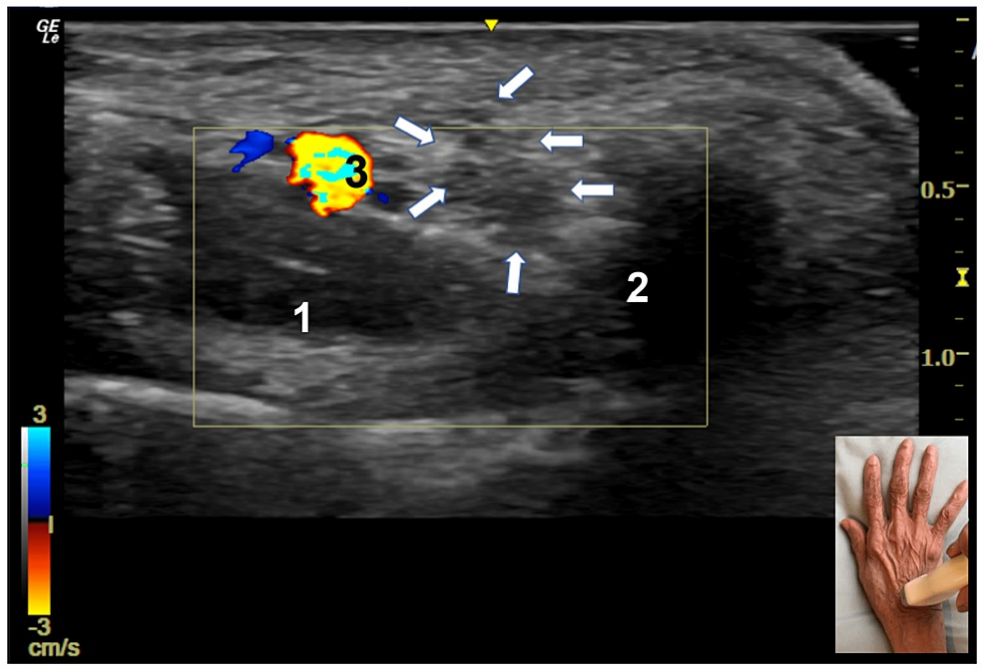
Ultrasound Study of the Distal Wrist Short axis view at the distal wrist showing the dorsal cutaneous branch of the ulnar nerve with prominent fascicles surrounded by hyperechoic tissue (arrows). The cross-sectional area of the DCBUN is 6 mm^2^ (normal ≤ 3 mm^2^). 1 and 2 are the 3rd and 4th dorsal interosseous muscles. A vein is depicted by 3. The scale for measuring the cross-sectional area is in mm^2^. DCBUN: dorsal cutaneous branch of the ulnar nerve

Of the two patients who underwent US studies, one had an increased CSA at the wrist with prominent fascicles and hyperechoic scar tissue (Figure 2), while the CSA was normal in the other patient.

Etiology

In 13 (92%) of the 14 cases, trauma was identified as the cause: iatrogenic in eight patients and due to external trauma in five. The iatrogenic causes included repair of torn ligaments in the wrist (three), ulnar shortening surgery (two), excision of a ganglion cyst (one), placement of a Scheker prosthesis after a fractured ulna (one), multiple surgeries for a triangular fibrocartilage complex (TFCC) tear, and wrist/forearm surgery from a wrist injury (one). The traumatic injury etiologies were as follows: motor vehicle accident (two), knife wound (one), dog bite (one), and a wrist laceration from a glass cut injury (one). No specific etiology was detected in one patient (7%).

## Discussion

**Figure 3:**
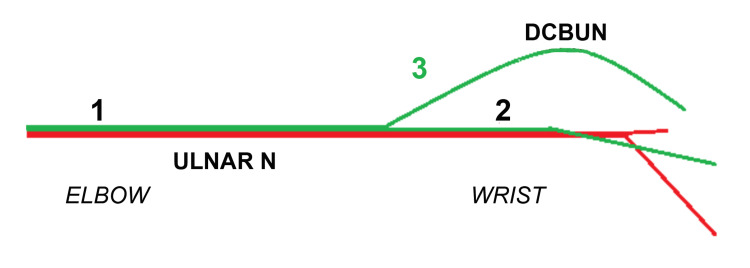
Localization of an Ulnar Nerve Injury 1. Elbow/proximal forearm 2. Wrist/Guyon’s canal 3. Dorsal cutaneous branch Red: Motor fibers to hypothenar and intrinsic hand muscles Green: Sensory fibers from DCBUN, palmar cutaneous nerve, and superficial ulnar nerve DCBUN: dorsal cutaneous branch of the ulnar nerve

A comprehensive clinical history and thorough neurological examination of the upper extremities are valuable in the evaluation of a patient with pain and altered sensation in the hand. Topography of sensory symptoms and signs limited to the dorsoulnar hand and dorsum of the fourth and fifth digits should suggest a DCBUN neuropathy and lead to comprehensive EDX studies of the upper extremities. While the EDX study provides an insight into the underlying pathophysiology (axon loss, demyelination, conduction block), the US study offers valuable information regarding the underlying pathology. Both of these tests together help to localize the lesion and reveal the cause [11,12,16,27]. Furthermore, EDX studies are crucial in differentiating distal ulnar nerve entrapment from proximal compression/entrapment at or near the elbow [12]. Young and colleagues studied EDX of the DCBUN in 27 asymptomatic patients (54 arms) and documented the average distal sensory latency and amplitude of the DCBUN [28]. These authors concluded that the distal sensory latencies were normal and stressed the importance of measuring and maintaining optimal arm temperatures to avoid inaccurate results [28]. The precise localization of distal ulnar nerve neuropathies depends upon findings from detailed EDX study of the ulnar nerve. In lesions proximal to the origin of the DCBUN, the SNAPs of digital branches and the DCBUN may be lost with additional motor conduction and EMG abnormalities. When the lesion is distal to the origin of the DCBUN, the findings include normal SNAP of the DCBUN with abnormal or absent ulnar digital SNAPs, prolonged distal motor latency, and EMG abnormalities in ulnar-innervated intrinsic muscles [12]. When there is selective involvement of the DCBUN, the SNAP is normal in the digital branch and absent in the DCBUN. Figure 3 depicts the localization of an ulnar nerve injury, and Table 2 highlights the EDX findings in an ulnar nerve neuropathy. 

**Table 2:** Electrodiagnostic Findings in an Ulnar Nerve Neuropathy * Location corresponds to the site of injury (1, 2, 3) in Figure 3 DCBUN: dorsal cutaneous branch of the ulnar nerve; SNAP: sensory nerve action potential; ADM: abductor digiti minimi; FDI: first dorsal interosseous; Abn: abnormal

Location *	SNAP of DCBUN	SNAP of Digital Branch	Motor Latency/Conduction Velocity	Denervation in ADM/FDI
1	Abn	Abn	Abn	Yes
2	Normal	Abn	Abn	Yes
3	Abn	Normal	Normal	No

US provides high-resolution images of the DCBUN and its relationship to adjacent structures [2,8]. US often accurately detects presence of entrapment neuropathy [4]. Not only is US an important test prior to orthopedic surgeries to document the origin and course of the DCBUN, but it is also essential postoperatively if an iatrogenic injury to the DCBUN occurs [8]. In addition to EDX and US studies in the assessment of a DCBUN injury, a regional anesthetic block of the DCBUN may also be useful to confirm the diagnosis [29]. Furthermore, an MRI of the distal forearm may be helpful to confirm or rule out lesions that may be compressing or entrapping the nerve [30]. 

To our knowledge, only six previous case reports/series have described injuries to the DCBUN (Table 3) [4,10,13,16,24,25]. Five of these previous reports highlight DCBUN injuries [4,10,16,24,25], while one reported an injury of the transverse radioulnar branch of the DCBUN [13]. Of the 15 previously reported cases, 12 (80%) were traumatic in nature, including four iatrogenic cases (surgery in the dorsoulnar aspect of wrist) and eight cases due to other traumatic injuries (blunt force and stretch injuries). Three patients sustained compression of the DCBUN, specifically, due to tenosynovitis of the extensor ulnar tendon in two cases and handcuff neuropathy in one case. Only two patients underwent EDX studies that revealed an absence of dorsal cutaneous response on the symptomatic side in one patient and a focal conduction block of the DCBUN in the other patient. Four patients had US studies that demonstrated a swollen and hypoechoic DCBUN. Our study concurs with the literature in that most cases of DCBUN neuropathy are traumatic, however, a higher percentage of our cases were iatrogenic. Similarly, most patients in our study showed an absence of DCBUN SNAPs. The iatrogenic cases illustrate the importance of identifying and protecting the DCBUN during surgery on the ulnar aspect of the wrist. 

**Table 3:** Injuries of the Dorsal Cutaneous Branch of the Ulnar Nerve in the Literature DCBUN: dorsal cutaneous branch of the ulnar nerve; SNAP: sensory nerve action potential; US: ultrasound; EDX: electrodiagnostic * Iatrogenic: repair of torn ligaments in the wrist (three), ulnar shortening surgery (two), excision of a ganglion cyst (one), Scheker prosthesis after a fractured ulna (one), multiple surgeries for a triangular fibrocartilage complex tear, and wrist/forearm surgery (one) ** Non-iatrogenic traumatic: motor vehicle accident (two), knife wound (one), dog bite (one), and a wrist laceration (one)

	Study	Number of Patients	Specific Injury	Additional Details
1	Lourie et al. [13]	3	Previous surgery in area of dorsoulnar area of wrist with postoperative dysesthesia and pain in ulnocarpal area; all 3 patients diagnosed with transection of transverse radioulnar branch of DCBUN	All 3 patients developed neuromas of the transverse radioulnar branch of DCBUN which were excised with improvement of dysesthesia
2	Robinson and Henderson [24]	1	Handcuff neuropathy of DCBUN (patient was professional drummer who had been handcuffed for 40 minutes)	EDX studies: absence of dorsal cutaneous response on symptomatic side, normal contralaterally
3	Grossman et al. [10]	6	Case 1: puncture wound to dorsum of right hand over 5^th^ metacarpal Case 2: open 5^th^ metacarpal fracture and extensor tendon injury Case 3: crush injury of hand Case 4: tenosynovitis of extensor carpi ulnaris tendon at level of ulnar head Case 5: puncture wound to dorsum hand Case 6: sustained saw injury, underwent open reduction and internal fixation of radial and ulnar fractures; DCBUN intact; physical therapy 4 months after injury, developed severe ulnar side wrist pain with paresthesia radiating to shoulder	Case 1: excised DCBUN neuroma with complete pain relief Case 2: excised DCBUN neuroma where it had been sutured to distal extensor tendon stump; 1 year f/u, symptom-free 1 year follow-up Case 3: EDX studies: focal conduction block of DCBUN; excised DCBUN neuroma; symptom-free Case 4: transcutaneous steroids by iontophoresis resolved symptoms Case 5: excised branch of DCBUN neuroma; symptom-free at 1 year follow-up Case 6: 6 mos after injury, underwent extensor tendolysis, compressed DCBUN released; symptom-free postoperatively
4	Chen et al. [25]	1	Direct injury to DCBUN caused by arthroscopic repair of triangular fibrocartilage complex; DCBUN was strangulated by one of the three pull-out sutures of the joint capsule, just ulnar to the extensor carpi ulnaris tendon	Pain and dysesthesia of ulnar aspect of wrist completely relieved after excision of injured nerve segment
5	Chang et al. [4]	1	Chronic extensor carpi ulnaris tenosynovitis with entrapment of DCBUN; the section of the entrapped DCBUN was proximal to the point giving off terminal branches, the ulnocarpal joint was underneath	US: DCBUN was swollen and hypoechoic proximal to thickened retinaculum, consistent with entrapment neuropathy; treated with low-intensity laser therapy with symptom improvement
6	Seo et al. [16]	3	Stretch and mild blunt injuries to DCBUN that occurred “during activities of daily living”	US: morphologic changes of DCBUN in all patients, including focal swelling with internal hypoechogenicity
7	Present Study 2023	14	Iatrogenic*: 8 (57%) Non-iatrogenic trauma**: 5 (36%) Idiopathic: 1 (7%)	EDX studies: SNAP of the DCBUN was not recordable in 13 (92%) patients; latency was increased and the amplitude was decreased in 1 patient who had a recordable SNAP with prolonged latency and low amplitude

The strength of the present study is that it includes the largest number of patients with DCBUN neuropathy confirmed by EDX studies. Our study has the potential to increase the awareness of DCBUN neuropathy in surgical settings and lessen the occurrence of iatrogenic injury. Limitations of our study include its retrospective nature and the lack of US studies in most cases. Another limitation is the lack of follow-up of patients after their EDX evaluation which has precluded our ability to assess the long-term outcome.

## Conclusions

Surgeons should be cognizant of the rare condition of DCBUN neuropathy and the many surgical procedures that may cause injury to this nerve. EDX and US studies are valuable in evaluating patients with clinical features of DCBUN neuropathy for confirmation of the diagnosis and determining the underlying etiology. Knowledge of the variations in the anatomy of the DCBUN preoperatively and incorporating precautionary measures during surgical procedures on the ulnar side of the wrist can potentially avoid inadvertent iatrogenic injuries. 
